# Optimizing psychotherapies for perinatal depressive symptom dimensions by strengthening social support networks: an exploratory mediation analysis approach

**DOI:** 10.1017/gmh.2024.116

**Published:** 2024-10-22

**Authors:** Ahmed Waqas, Siham Sikander, Abid Malik, Najia Atif, Atif Rahman

**Affiliations:** 1 Department of Primary Care and Mental Health, Institute of Population Health, University of Liverpool, Liverpool, UK; 2 Human Development Research Foundation, Islamabad, Pakistan; 3 Department of Public Mental Health, Health Services Academy, Islamabad, Pakistan

**Keywords:** mediation analysis, CBT, cognitive behavioral, perinatal depression, perinatal anxiety, transdiagnostic, social support, stratified psychotherapy

## Abstract

The Thinking Healthy Program (THP) is a multicomponent low-intensity cognitive behavioral therapy-based psychosocial intervention. This intervention has been shown to be clinically effective in perinatal depression (PND) and feasible for implementation in low-resourced settings. It has also been shown to work universally for different phenotypes of PND. However, the mechanism through which THP resolves different phenotypes of PND are unclear. The present investigation presents secondary mediation analyses of a dataset curated from a cluster randomized controlled trial conducted in Pakistan assessing the effectiveness of the THP. Women aged 16–45 years in their third pregnancy trimester, with a diagnosis of PND, underwent 16 sessions of the intervention. The severity of depression was assessed using the Hamilton Depression Rating Scale (HDRS). 2-1-1 mediation models revealed that social support exerted significant mediation in driving the intervention effects for improving the severity of depressive symptoms on the HDRS [*B* (SE) = 0.45 (0.09), 95% CI: 0.30–0.60] and its symptom dimensions of core emotional symptoms [*B* (SE) = 0.27 (0.06), 95% CI: 0.18–0.37], somatic symptoms [*B* (SE) = 0.24 (0.04), 95% CI: 0.16–0.31] and insomnia symptoms [*B* (SE) = 0.04 (0.02), 95% CI: 0.02–0.07].

## Impact statement

The Thinking Healthy Program (THP) is a cognitive-behavioral-based psychosocial intervention endorsed by the World Health Organization for treating perinatal depression (PND). As part of the WHO’s Mental Health Gap Action Program, the THP aims to expand access to services for perinatal mental health, particularly in low- and middle-income countries.

Currently, the program is undergoing innovative adaptations, including cross-cultural modifications and trials in several countries, to be delivered as a digital health intervention and for the prevention of PND to enhance infant well-being. Despite its widespread use, there is limited research on the mechanisms through which psychosocial interventions like THP produce their effects, and for which populations they are most effective – a critical research question that has puzzled scientists over the past decade.

Understanding these mechanisms is essential for optimizing psychosocial treatments. This study specifically investigates social support as a key factor contributing to the effectiveness of THP and examines whether social support can lead to improvements across different symptom dimensions of PND.

## Background

Perinatal depression (PND) is a major global mental health concern due to its high prevalence and negative maternal and infant health consequences (Atif et al., 2021; Fisher et al., 2012; Gelaye et al., 2016). Its prevalence has been estimated to be as high as 37% in countries like Pakistan (Atif et al., 2021), where it is an established risk factor for poor maternal psychosocial functioning, marital problems, medical comorbidities and infant illnesses like increased episodes of diarrheal illness, respiratory infections and poor growth and neurodevelopment (Gelaye et al., 2016; Naveed et al., 2018; Rahman et al., 2004; Waqas et al., 2015, 2018). Recognizing these deleterious consequences of PND, interdisciplinary research has gained momentum to tackle this public health issue. This body of research includes understanding the nature of PND, its subtypes and pathophysiology (Guintivano et al., 2019; Juruena et al., 2018; Putnam et al., 2015, 2017), screening measures and pharmacological and psychological prevention measures and treatments (Rahman et al., 2018; Singla et al., 2021; Waqas et al., 2021a; Wilson et al., n.d.). Among the treatment choices available for PND, psychological therapies have been found to be effective clinically, with little to no associated harm (Rahman et al., 2013, 2018). Several strategies have been suggested to improve equitable access to these treatments including by task shifting and employing technology for training, supervision and delivery (Rahman et al., 2021).

Recent research on PND has indicated that it is a heterogeneous disorder (Putnam et al., 2017). Investigations using different statistical methods have been published to understand this heterogeneity. For example, data-driven subtyping of PND, latent class analyses and growth curve models, and more recently, symptom networks have been used to understand different subtypes of PND and its trajectories overtime (Mora et al., 2009; Santos et al., 2017a, 2017b; Sun et al., 2020; Yu et al., 2020). Studies using growth mixture models have revealed varying trajectories based on time of onset of symptoms and their longitudinal trends based on the severity of depression, yielding two to six different trajectories (Santos et al., 2017a). Latent class and cluster analyses have focused on symptom-specific heterogeneity. Putnam and colleagues (Putnam et al., 2015, 2017) used data from the Postpartum Depression: Action Toward Causes and Treatment consortium and employed cluster analyses to reveal five distinct classes of depression with varying symptom qualities, namely: severe anxious depression, moderate anxious depression, anxious anhedonia, pure anhedonia and resolved depression (Putnam et al., 2017). Using latent class analyses, they reported three distinct classes of PND, with striking differences in severity of PND (Putnam et al., 2015). The class of PND with most severe depression presented with mixed depression and anxiety symptoms, reported suicidal ideation and had an onset during pregnancy. The next class with middling severity of PND had onset in 4 weeks postpartum and had more pregnancy-related complications, while the third class had the least severe depression scores and pregnancy complications (Putnam et al., 2015). These results were also corroborated by our recent investigation reporting four distinct classes of PND: mixed anxious depression, somatic depression, atypical depression and mild depression (Waqas & Rahman, 2021b). There is little research, however, exploring the differential responses of treatments (if any) to different subtypes of depression.

In treatment research for PND, psychological therapies for PND have shown the most promise (Rahman et al., 2013, 2018). An increasing body of research has shown that these treatments work both in high- and low-resourced settings. However, there is a lack of research evidence on how and for whom these interventions work (Huibers et al., 2021). Delineating mechanistic pathways of different treatments have gained priority in the research agenda for depression (Huibers et al., 2021), so that treatment approaches can be optimized.

Research in delineating the ideal active components or the causal processes of change in treatment for PND, noted for its heterogeneous nature, are still lacking (Abraham and Michie 2008, Chorpita et al. 2005, Chowdhary et al. 2014, Cuijpers et al. 2019, Lipsitz and Markowitz 2008, Paul 1967, Singla et al. 2014). Previous meta-analytical investigations point out that effect sizes across different psychotherapies are comparable and that multicomponent therapies consisting of both specific and nonspecific components are more effective (Rahman et al., 2018). An important nonspecific ingredient used in treatment of PND is social support which can broadly be classified into identifying sources of social support, activating existing social networks or forming new ones (Taylor, 2011). Social support has been found to be an active component in several successful programs, such as the THP tested on a large scale in Pakistan (Rahman et al., 2008) and India (Huang et al., 2020). A recent meta-analysis reported moderate effects of peer support interventions on PND, after pooling effect sizes across 10 studies from different countries (Huang et al., 2020).

Social support during pregnancy pertains to the perinatal women’s quality of human relationships and the degree of interconnectedness with the community and environment (Lakey and Cohen 2015, Sharif et al., 2021). Social support is essentially a multidimensional construct, the definition of which varies with the theoretical or contextual framework being applied (Taylor, 2011; Waqas et al., 2015). One definition is based on the source of support such as from the friends, family and significant others (Sharif et al., 2021). Another definition takes into account the functional aspect of support offered, such as informational, instrumental or emotional support (Taylor, 2011). Regardless of the theoretical frameworks being applied, social support has been consistently shown to be a strong preventive factor and active component of psychotherapeutic interventions for PND (Biaggi et al., 2016; Rahman et al., 2008). Several successful interventions have used social support as a treatment strategy, including the THP delivered by lay health workers and peers (Maselko et al., 2020; Rahman et al., 2008); Gao et al.’s (2010) interpersonal psychotherapy-oriented childbirth education program, and; Tripathy et al.’s (2010) participatory action cycle. A recent systematic review of psychotherapeutic interventions for PND showed that 15 out of 33 interventions had social support as a treatment component (Rahman et al., 2018).

The present investigation aims to shed light on two aspects: exploring the role of social support as a treatment component of a multicomponent intervention and matching it to subtypes of PND that respond to it. We essentially explore the mediational pathways of social support, through which this low-intensity multicomponent cognitive behavioral-based intervention program works in improving PND. These mechanistic pathways are explored from the lens of stratified psychiatry, building upon our previous work on data-driven subtypes of PND (Waqas & Rahman, 2021b). We consider several subtypes of PND, as a product of differing severity scores on different symptom dimensions of mixed anxiety and depression symptoms, somatic symptoms, insomnia symptoms and atypical symptoms (Waqas & Rahman, 2021b). We explore how social support works, and for whom, in a sample of perinatally depressed women who received the intervention.

## Methods

### Study design

The present investigation presents secondary mediation analyses of a dataset curated from a cluster randomized controlled trial (cRCT) assessing the effectiveness of a task-shifted multicomponent cognitive behavioral therapy-based program, conducted in Pakistan. Details on the study design can be found in previous publications (Waqas & Rahman, 2021b; Rahman et al., 2008). Briefly, this program was tested in two rural subdistricts of Rawalpindi; Gujar Khan and Kallar Sayedan, which are homes to over 750,000 Punjabi and Potohari populations. During the study period, the majority of families in these areas relied on subsistence farming, with additional income typically generated by men who served in the military, worked as government employees, or performed semiskilled or unskilled labor positions in urban areas. Primary care in these areas is provided by Basic Health Units, which employ teams of primary care doctors and the allied health workers. Among allied clinical staff, teams of lady health workers (LHWs) provide primary obstetric care and childcare in adjacent communities.

The analyses in this study are based on data from a cRCT conducted between April 2005 and March 2007 that evaluated the THP. This dataset is of enduring relevance as it provided significant evidence of the effectiveness of a cognitive-behavioral therapy (CBT)-based interventions delivered by LHWs. The robust treatment effects observed in this trial were pivotal in the World Health Organization’s (WHO) decision to endorse the THP as a model low-intensity psychosocial intervention for PND within its Mental Health Gap Action Program (Rahman et al., 2013, 2018).

For recruitment in the trial, the LHWs identified all pregnant women aged 16–45 years in their third trimester of pregnancy, who were residents of 40 union councils in the study area. These 40 union councils were randomized to intervention and control clusters; using a table of random numbers by a researcher who was not involved in the study and had no knowledge of the identities of the Union Councils.

Consenting women were offered clinical assessment for PND using the Structured Clinical Interview for DSM-4 Depressive Episode, and symptom severity was rated using the Hamilton Depression Rating Scale (HDRS). Those diagnosed with depression were invited to participate in the trial. Those with serious medical and psychotic illnesses requiring hospitalization were excluded. Consenting women were enrolled in the trial and were given either the intervention or care as usual according to their allocation status. Written informed consent was received from all the participants by study research staff and ethical approval was sought and provided by the University of Manchester, UK, and the Institute of Psychiatry, Rawalpindi, Pakistan. Further details on the selection of participants are provided elsewhere (Rahman et al., 2008).

### Social support modules in the THP

The intervention recipients received the multicomponent low-intensity cognitive behavioral intervention from their respective LHWs (two in each cluster) who were trained in the THP. The THP comprised of 16 sessions, each lasting from 45 min to 1 h. The intervention started in the last trimester of pregnancy, with one weekly session for 4 weeks, three fortnightly sessions during the first two postnatal months, and nine monthly sessions up until the tenth postnatal month (World Health Organization, 2015). In each session, the specific ingredients based on the principles of cognitive behavior therapy included guided discovery using illustrations to break the cycle of maladaptive thoughts and actions, and behavioral activation and problem solving to gently encourage the depressed participant become more active in her day-to-day activities. Nonspecific ingredients included empathetic listening by the health worker and harnessing support from key family members to enhance the level of social support received by the woman.

This section provides details on the social support strategies employed in the THP. Details on other treatment components of THP especially the CBT are provided in the THP field manual published by the World Health Organization in six languages (World Health Organization, 2015). In the five sessions where social support was assessed, standardized guidelines for empathetic listening and communication were applied. The THP emphasizes the importance of empathy and effective communication as key components of the intervention. LHWs were trained to listen attentively, show understanding, and engage with the mothers in a respectful manner, ensuring that the mothers felt comfortable, trusted and supported. Specific guidelines included techniques such as active listening, open-ended questioning, summarizing the mother’s concerns and maintaining a positive and nonjudgmental approach throughout the interactions. These practices were integral to fostering a therapeutic relationship and enhancing the perceived social support provided by the LHWs (World Health Organization, 2015).

**Figure 1.**
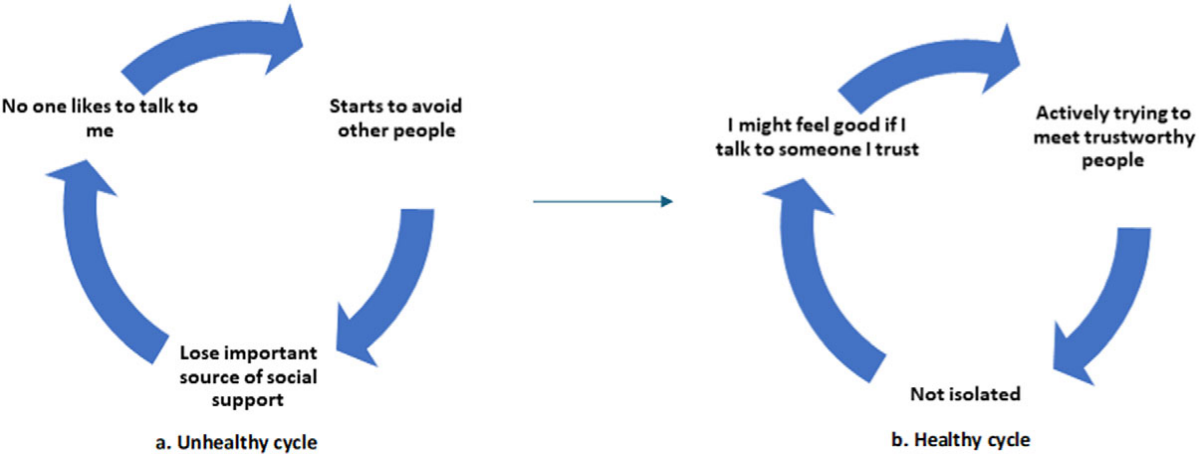
An example of cognitive behavioral strategies for improving social support.

Content related to social support was embedded in each module of the THP. The delivery agents of the THP were trained in empathic listening skills and involving key family members to encourage reactivation of social support networks. Five of the sixteen sessions focused specifically on the woman’s relationship with significant others and harnessing their support. A two-pronged strategy was used to enhance the levels of social support. First, the participating women were advised to keep a mood chart about unhelpful thoughts related to people around them. After psychoeducation about the importance of social support in good quality care, pictorial illustrations were used to illustrate unhelpful thoughts about people around the mother. The women were taught to identify unhelpful thoughts such as “people do not care about me” and “I can’t be bothered to meet my friends”. Using the technique of ‘normalization’, the women were helped to understand that they are not alone in experiencing such thoughts and how this often led to social withdrawal and loss of important sources of support. Such thoughts were gently challenged, healthy alternatives were suggested, and engagement in social activities was encouraged. Second, the health worker engaged key family members of the women, including the husband and in-laws. They were educated about the importance of social support for the mother’s welling during prenatal and postnatal periods and its significance for the baby. An example of the mood chart on social support, adapted from the THP manual, is presented in Figure 1.

Special charts were used to assess and monitor the women’s social support networks. Family members were advised to identify stressors during the perinatal period and to help the woman cope with these. They were educated about the benefits of social support in the healthy development of the baby. The health workers also encouraged women to form a peer group with other expecting women in the locality.

### Treatment as usual

Mothers in the control cluster received the same number of visits as those in the intervention group, conducted in a similar manner by routinely trained LHWs (two per Union Council). Both groups of health workers were supervised monthly and monitored by the research team to ensure they completed the scheduled visits.

### Measures

The trial participants were interviewed by two qualified psychiatrists who were blind to their allocation status. Interviews were done at three time points: baseline, 6 months postnatal and 12 months postnatal. A battery of validated assessment measures in local languages was employed, including participant characteristics, the HDRS and the Multidimensional Scale of Perceived Social Support (MSPSS), in addition to other maternal and child health-related variables. For this study, we utilized data pertaining to the HDRS and MSPSS.

There were four outcomes for this investigation yielded from the HDRS. These included the total scores on the HDRS as the severity of depressive symptoms and three symptom dimensions as delineated in our previous publication (Waqas & Rahman, 2021b). The three symptom dimensions included core emotional symptoms, symptoms of insomnia and somatic symptoms. The core emotional symptoms comprised of symptoms of sad mood, anhedonia, loss of appetite, somatic anxiety and psychic anxiety. Symptoms of insomnia included insomnia during early night and middle and late periods. The last symptom dimension comprised of somatic symptoms of weight loss, psychomotor retardation, somatic symptoms, hypochondriasis and suicidal ideation. These symptom dimensions were derived from the data using principal component analyses, yielding adequate communalities (>0.2) and factor loadings (>0.4). Detailed analyses are presented elsewhere (Waqas & Rahman, 2021b).

The mediating variable in this investigation was perceived social support, which was assessed using the Urdu version of the MSPSS scale (Akhtar et al., 2010). It assesses the levels of perceived social support from family, friends and significant others. The Urdu version of this scale has been previously validated and found reliable for use in Pakistan (Sharif et al., 2021). It comprises 12 items, with 4 items corresponding to each subscale. Sample statements include “There is a special person who is around when I am in need”. Responses are recorded on a 7-point Likert scale ranging from “very strongly disagree” to “very strongly agree”.

### Analyses

All analyses were conducted in SPSS (v. 25) and MPlus (v 7.0). Quantitative variables were presented as mean (SD) and categorical variables as frequency (%). The present investigation presents secondary mediation analyses within the context of a cRCT where participants were resident in specific geographical areas (Union Councils) randomized to THP intervention or enhanced usual care. Due to the hierarchical nature of the dataset, the mediation analyses were conducted using multilevel models. A series of multilevel models were run to assess the suitability of the data for running mediation analyses. The assumptions for mediation analysis were met if linear mixed-effects regression demonstrated a statistically significant association between the independent variable and the mediator and between the mediator and outcome (Baron & Kenny, 1986).

The mediation analyses were conducted in a multilevel structural equation modeling framework using the MPlus software (version 8). For this, we tested a 2-1-1 mediation model, where mediation was hypothesized at the level of clusters. This model was found to be appropriate because the intervention condition was randomized at the level of clusters rather than individuals. The mediator variable and social support scores were available at the level of individuals and transformed into cluster-level mean scores to model higher-level variance and group mean centered scores for lower-level variance. Outcome variable pertained to scores for three dimensions of HDRS, which were decomposed into latent within- and between-level parts.

## Results

There were 903 participants across 40 Union Councils, of which 440 participants were in the control cluster and 463 in the intervention cluster. The average age of the participants was 26.74 (5.11) years. Complete data were available for 818 participants (90.59%) at the sixth month follow-up, of which 418 were in the intervention group and 400 were in the control group. No significant differences in age, education or SES were found among the completers and dropouts. Out of 818 participants, the intervention and control groups reported no significant differences in age, socioeconomic class, spouse’s education levels, severity of depression and disability, and social support scores at baseline. Participants in the intervention group had slightly higher education levels (Table 1).

**Table 1.** Baseline characteristic of trial participants

Variable	THP arm (*n* = 418)	Control arm (*n* = 400)
Maternal age	26.49 (5.06)	27 (5.16)
Maternal education^†^	4.36 (4.03)	3.76 (3.88)
Father’s education	6.94 (4.01)	7.06 (3.93)
Number of children^†^	2.15 (1.75)	2.40 (1.79)
HDRS	14.71 (4.17)	14.43 (3.96)
BDQ	8.09 (2.72)	8.28 (2.67)
MSPSS	45.77 (16.45)	44.55 (16.10)
Socioeconomic class	3.58 (0.96)	3.65 (0.97)

† *p* < 0.05.

HDRS, Hamilton Depression Rating Scale; BDQ, Brief Disability Questionnaire; MSPSS, The Multidimensional Scale of Perceived Social Support.

Main effects analyses at 6-month follow-up showed that participants in the intervention group exhibited lower severity of depressive symptoms on the HDRS [*B* (SE) = −4.08 (0.78), *p* < 0.001] and its symptom dimensions of core emotional symptoms [*B* (SE) = −2.31 (0.61), *p* < 0.001], somatic symptoms [*B* (SE) = −1.0 (0.15), *p* < 0.001] and insomnia symptoms [*B* (SE) = −0.56 (0.14), *p* < 0.001], compared to the control group. The intervention group also showed improved scores on the social support scale [*B* (SE) = 6.93 (1.56), *p* < 0.001], which in turn was associated with lower HDRS scores [*B* (SE) = −0.21 (0.01], *p* < 0.001), and its symptom dimensions of core emotional symptoms [*B* (SE) = −0.10 (0.01), *p* < 0.001], somatic symptoms [*B* (SE) = −0.04 (0.003), *p* < 0.001] and insomnia symptoms [*B* (SE) = −0.04 (0.003), *p* < 0.001]. The results showing mean differences for intervention and control groups across the variables are summarized in Table 2. Detailed statistics on effectiveness of the interventions in improving HDRS, and social support and three symptom dimensions are described elsewhere (Rahman et al., 2008; Waqas & Rahman, 2021b).

**Table 2.** Scores on the social support and depression scale at 6-month follow-up

Variables	THP	Control	Mean difference (THP-control)^†^
	Mean (SD)	Mean (SD)	
Depressive symptoms	4.48 (5.99)	8.68 (7.40)	−4.20^†^
Core emotional symptoms	2.53 (3.40)	4.935 (4.28)	−2.40^†^
Insomnia symptoms	0.54 (1.38)	1.11 (1.72)	−0.57^†^
Somatic symptoms	1.07 (1.50)	2.08 (1.90)	−1.01^†^
Social support scores	50.75 (15.32)	43.69 (15.67)	7.06^†^

† *p* < 0.001.

2-1-1 mediation models (Table 3) revealed that social support exerted significant mediation in driving the intervention effects for improving the severity of depressive symptoms on the HDRS [*B* (SE) = 0.45 (0.09), 95% CI: 0.30–0.60] and its symptom dimensions of core emotional symptoms [*B* (SE) = 0.27 (0.06), 95% CI: 0.18–0.37], somatic symptoms [*B* (SE) = 0.24 (0.04), 95% CI: 0.16–0.31] and insomnia symptoms [*B* (SE) = 0.04 (0.02), 95% CI: 0.02–0.07].

**Table 3.** 2-1-1 mediational effects of social support on depression scores

		Direct effects at cluster and individual level (*B*, SE)		Indirect effects (*B*, 95% CI)
Depression scores	Level of measurement	MSPSS	Thinking Healthy Program	
Total scores	Level 1	−0.203 (0.017)^2^	–	
	Level 2	−0.397 (0.047)^2^	−0.423 (0.179)^3^	0.45 (95% CI: 0.30–0.60)
Core emotional symptoms	Level 1	−0.10 (0.01)^1^	–	
	Level 2	−024 (0.035)^2^	−0.556 (.07)^2^	0.27 (95% CI: 0.18–0.37)
Insomnia symptoms	Level 1	−0.039 (0.004)^2^	–	
	Level 2	−0.039 (0.011)^2^	−0.148 (0.039)^2^	0.044 (95% CI: 0.02–0.07)
Somatic symptoms	Level 1	−0.05 (0.005)	–	
	Level 2	−0.207 (0.018)^2^	2.146 (0.091)^2^	0.235 (95% CI: 0.162–0.307)

Cluster mean scores were used in structural equation models to model relationships at cluster level and group mean centered scores at individual level.

1 *p* < 0.05.

2 *p* < 0.01.

3 *p* < .001.

Level 1 corresponds to individual level and level 2 at cluster level.

## Discussion

The present investigation explores the potential of enhancing social support as an active component in a psychotherapeutic intervention for PND. A novel aspect of the exploratory mediation analyses is the consideration of the effects of social support on different symptom dimensions of PND. Our analyses revealed that strategies for enhancing social support yield a positive effect on all the three different dimensions of PND, namely somatic symptoms, anxiety and mood symptoms and symptoms of insomnia. This indicates that it is a valuable treatment component that should be included in psychotherapeutic programs.

This work builds on our previous study, which indicated that PND is a heterogeneous condition (Waqas & Rahman, 2021b). We identified three different symptom dimensions of PND, including somatic symptoms, anxiety and mood symptoms and symptoms of insomnia, among perinatal women in Pakistan. Evaluating the impact of individual ingredients of a multicomponent intervention on each of these dimensions could potentially assist in refining treatments to individual needs. Stratified psychiatric approaches have recently generated significant attention among mental health researchers (Huibers et al., 2021). Individual Patient Data meta-analytical approaches are increasingly being used to identify different treatment elements to match with different subtypes of depression [(Furukawa et al., 2021; Pompoli et al., 2018). Although research toward developing such stratified treatments in psychiatry is essential, such approaches are not always feasible in low-resourced settings. As opposed to HICs, LMICs neither have the financial resources nor human personnel to implement extensive stratified psychotherapeutic approaches requiring specialist delivery (World Health Organization, 2008), after evaluating and matching different patients to a suitable treatment. Therefore, it is crucial to identify nonspecific approaches, such as social support that can be applied with relative ease by non-specialists and universally benefit perinatal women with different subtypes of depression (Waqas & Rahman, 2021b).

In the present analyses, we report a simple pathway where the Thinking Healthy intervention improves depressive symptoms indirectly through perceived improvements in self-reported social support levels. As detailed previously, social support is multidimensional and may itself have multiple pathways through which it leads to a decrease in depressive symptoms; for example, by activating social networks to increase opportunities for receiving quality healthcare; provision of material and emotional support; or even by reducing the levels of inflammatory biomarkers (Singla et al., 2019). However, due to limitations of the available data, we could not test these complex serial mechanisms and pathways. Furthermore, there may be interactions between active ingredients of the multicomponent intervention translating into more complex mediational pathways. For example, Thinking Healthy employs cognitive behavioral strategies to challenge negative cognitive schema pertaining to one’s social network, and then to “activate” the perinatal women to reengage with their social network. This might create a synergistic relationship between cognitive restructuring, behavioral activation and social support, leading to the reduction in depressive symptoms. This is also corroborated in another study where THP was delivered by peers in India and Pakistan, where mediation analyses on the pooled data showed that behavioral activation was one of the mediational mechanisms for improving depressive symptoms in the perinatal women (Singla et al., 2019).

Similarly, encouraging family members to play an active role in childcare support may have led to better emotional and tangible support for the woman (Rahman et al., 2008). The data show that the incidence of diarrhea was reduced, and the rates of immunization and frequency of parental time spent playing with the infant were increased in women who received the intervention (Rahman et al., 2008). The interplay of all these variables in producing the desired outcomes in both mothers and infants is more likely, rather than a single ingredient playing a major role. Our findings are corroborated by other studies that employ multiple strategies, such as provision of emotional, informational and tangible support and opportunities for empathic listening and catharsis through nondirective counseling, leading to improvement in emotional or cognitive symptoms of depression (Morrell et al., 2009).

An important finding in our analyses was improvement in somatic symptoms among perinatal women, mediated through social support. In our study sample, a significant percentage of women reported depression with the somatic phenotype. Previous studies have demonstrated that recognition of depression is difficult in primary care for patients reporting with somatic symptoms of depression (Kapfhammer, 2006; Tylee & Gandhi, 2005). Furthermore, somatic symptoms can also complicate the treatment of depression. Tylee and Gandhi demonstrated that the number of somatic symptoms is a risk factor for treatment resistance and poor treatment response in the treatment of major depression, where patients with severe aches and pains at baseline are about four times less likely to report poor treatment response (Tylee & Gandhi, 2005). Therefore, it is important to consider suitable treatment approaches for this subgroup of perinatal women with depression and enhancing social support may be a feasible strategy. There is evidence on the clinical utility of social support enhancing strategies for somatic complaints in major depressive disorder in the general population (Das et al., 2020; Grigaitytė & Söderberg, 2021a), but more research is needed in the perinatal population.

Other hypothesized pathways through which social support mediates improvement in depressive symptoms are through increased emotional self-efficacy and regulation in individuals (Cohen and Wills 1985, Grigaitytė & Söderberg, 2021a). Although evidence is scarce, social support might also improve symptoms, especially somatic, by reducing inflammation and improving HPA-axis function (Kapfhammer, 2006; Uchino et al., 2018). More research is needed to elucidate these mechanisms fully.

## Direction for future work

Our findings highlight the critical role of social support as a potent active ingredient in the THP. The current strategies within THP, including cognitive restructuring and behavioral activation, have been effective in helping participants identify sources of social support, strengthen their social networks and engage more deeply with LHWs. This approach underscores the significance of social support in enhancing the overall effectiveness of the intervention. However, there are opportunities to further amplify this aspect of THP. One potential enhancement could involve embedding community-building activities within the intervention. This might include incorporating peer support groups, and offering group therapy sessions, to extend the reach of social support (Husain et al., 2022). By integrating these strategies, the intervention could more effectively target social engagement, which appears to be a crucial pathway to achieving desirable outcomes.

Additionally, an additional module focused explicitly on strengthening social support could be introduced to reinforce these efforts and ensure that this vital component of the intervention is maximized. These strategies could also be extended to digital mental health therapies by creating virtual support groups and fostering digital communities where participants can engage in shared experiences and receive peer support. Integrating social support in digital mental health therapies could boost the overall effectiveness of the intervention in a scalable and accessible way, an exciting avenue currently being explored in rural Pakistan (Atif et al., 2022).

## Strengths and limitations

This investigation has several strengths, including a large sample size and rigorous data collection procedures embedded in a pragmatic RCT. Furthermore, the focus on symptom dimensions, which accounts for covert heterogeneity in depression, is another strength of this study. Mediation analyses were conducted using a multilevel framework, which appreciates the nested structure of the data yielded from a cRCT. Women included in this study underwent DSM-IV psychiatric diagnoses for PND, which reduces the concerns related to reliability and validity seen in studies employing psychometric scales for screening or measuring severity of PND. The main limitation is the lack of more detailed objective data on the various dimensions of social support. In addition, we do not have data on biological markers of PND, such as the levels of inflammatory and immunological biomarkers, and levels of hormones secreted by the hypothalamus and pituitary gland during pregnancy.

A key limitation of our study is the potential oversight of more intricate mediational pathways that were not explored. Important mediators, such as empathy, the quality of the patient–therapist relationship, patient activation, problem solving and cognitive restructuring, have been highlighted in the literature as critical components in similar therapies (Singla et al., 2017). Their exclusion from our analysis may have led to an incomplete understanding of the full range of mediational influences within the THP. To address this limitation, future studies should incorporate these hypothesized mediators; to gain a more comprehensive understanding of the mechanisms through which THP exerts its therapeutic effects. The lack of such data hindered exploration of more complex serial mediational pathways between the THP intervention and the symptom dimensions of depression. Researchers are encouraged to explore these pathways in their future studies.

## Data Availability

All data related to the manuscript are available upon request.
