# Supplementary material for: Optimizing psychotherapies for perinatal depressive symptom dimensions by strengthening social support networks: an exploratory mediation analysis approach
Source: Glob Ment Health (Camb). 2024 Oct 22;11:e91. doi: 10.1017/gmh.2024.116 (PMC11504941; doi:10.1017/gmh.2024.116)
Supplement: Peer review history

# Editor report: Recommendation: Optimizing psychotherapies for perinatal depressive symptom dimensions by strengthening social support networks: an exploratory mediation analysis approach — R0/PR2

**DOI:** 10.1017/gmh.2024.116.pr2

**Affiliations:** Fudan University, China

No accompanying comment.

# Editor report: Decision: Optimizing psychotherapies for perinatal depressive symptom dimensions by strengthening social support networks: an exploratory mediation analysis approach — R0/PR3

**DOI:** 10.1017/gmh.2024.116.pr3

**Affiliations:** Johns Hopkins University Bloomberg School of Public Health, United States

No accompanying comment.

# Editor report: Recommendation: Optimizing psychotherapies for perinatal depressive symptom dimensions by strengthening social support networks: an exploratory mediation analysis approach — R1/PR5

**DOI:** 10.1017/gmh.2024.116.pr5

**Affiliations:** Fudan University, China

No accompanying comment.

# Editor report: Decision: Optimizing psychotherapies for perinatal depressive symptom dimensions by strengthening social support networks: an exploratory mediation analysis approach — R1/PR6

**DOI:** 10.1017/gmh.2024.116.pr6

**Affiliations:** Johns Hopkins University Bloomberg School of Public Health, United States

No accompanying comment.
